# Determining the spatial distribution of environmental and socio-economic suitability for human leptospirosis in the face of limited epidemiological data

**DOI:** 10.1186/s40249-022-01010-x

**Published:** 2022-08-04

**Authors:** Maximiliano A. Cristaldi, Thibault Catry, Auréa Pottier, Vincent Herbreteau, Emmanuel Roux, Paulina Jacob, M. Andrea Previtali

**Affiliations:** 1 Department of Natural Sciences, College of Humanities and Sciences, National University of Litoral, Santa Fe, Argentina; 2 ESPACE-DEV, French National Research Institute for Sustainable Development (IRD), University of Montpellier, University of French West Indies, University of French Guiana, University of La Reunion, Montpellier, France; 3 Sentinela International Joint Laboratory, French National Research Institute for Sustainable Development (IRD), University of Brasilia (UnB), Oswaldo Cruz Foundation (Fiocruz), Brasília, Brazil; 4 Sentinela International Joint Laboratory, French National Research Institute for Sustainable Development (IRD), University of Brasilia (UnB), Oswaldo Cruz Foundation (Fiocruz), Rio de Janeiro, Brazil; 5 National Institute of Respiratory Diseases (INER) “Dr. E. Coni”/National Administration of Health Institutes (ANLIS “Dr. C.G. Malbrán”), Santa Fe, Argentina; 6 Leptospirosis Laboratory, College of Biochemistry and Biological Sciences, National University of Litoral, Santa Fe, Argentina; 7 National Scientific and Technical Research Council (CONICET), Santa Fe, Argentina

**Keywords:** Spatial epidemiology, Underreported misdiagnosed diseases, Environmental conditions, Socioeconomic groups, Knowledge-based index, Cluster analysis

## Abstract

**Background:**

Leptospirosis is among the leading zoonotic causes of morbidity and mortality worldwide. Knowledge about spatial patterns of diseases and their underlying processes have the potential to guide intervention efforts. However, leptospirosis is often an underreported and misdiagnosed disease and consequently, spatial patterns of the disease remain unclear. In the absence of accurate epidemiological data in the urban agglomeration of Santa Fe, we used a knowledge-based index and cluster analysis to identify spatial patterns of environmental and socioeconomic suitability for the disease and potential underlying processes that shape them.

**Methods:**

We geocoded human leptospirosis cases derived from the Argentinian surveillance system during the period 2010 to 2019. Environmental and socioeconomic databases were obtained from satellite images and publicly available platforms on the web. Two sets of human leptospirosis determinants were considered according to the level of their support by the literature and expert knowledge. We used the Zonation algorithm to build a knowledge-based index and a clustering approach to identify distinct potential sets of determinants. Spatial similarity and correlations between index, clusters, and incidence rates were evaluated.

**Results:**

We were able to geocode 56.36% of the human leptospirosis cases reported in the national epidemiological database. The knowledge-based index showed the suitability for human leptospirosis in the UA Santa Fe increased from downtown areas of the largest cities towards peri-urban and suburban areas. Cluster analysis revealed downtown areas were characterized by higher levels of socioeconomic conditions. Peri-urban and suburban areas encompassed two clusters which differed in terms of environmental determinants. The highest incidence rates overlapped areas with the highest suitability scores, the strength of association was low though (CSc *r* = 0.21, *P* < 0.001 and ESc *r* = 0.19, *P* < 0.001).

**Conclusions:**

We present a method to analyze the environmental and socioeconomic suitability for human leptospirosis based on literature and expert knowledge. The methodology can be thought as an evolutive and perfectible scheme as more studies are performed in the area and novel information regarding determinants of the disease become available. Our approach can be a valuable tool for decision-makers since it can serve as a baseline to plan intervention measures.

**Supplementary Information:**

The online version contains supplementary material available at 10.1186/s40249-022-01010-x.

## Background

Leptospirosis is a disease caused by a bacterium of the genus *Leptospira* and is among the leading zoonotic causes of morbidity and mortality worldwide [1]. Although humans can be exposed directly or indirectly to pathogenic leptospires, indirect transmission through contact with an environment contaminated with the bacteria is the most frequent human exposure route [2, 3]. Since the transmission dynamics of leptospires depends on interactions between human beings, reservoirs (mainly mammals), and the environment, human infections are strongly associated with ecological and local socioeconomic determinants [4, 5]. Higher incidence is reported in tropical, humid, and temperate regions, especially during the warmer and wetter months [3, 4]. Heavy rainfall and flooding often trigger leptospirosis outbreaks by increasing human contact with animal hosts, contaminated water, and mud [4, 5]. Leptospirosis commonly occurs in rural areas affecting the most marginalized populations such as rural subsistence farmers [5, 6]. However, leptospirosis outbreaks are now increasingly reported in urban slums of developing countries where infection is often associated with inadequate sanitation and poverty [4]. Leptospirosis has significant health and economic consequences for affected patients and countries [7, 8]. Prevention and early case detection and treatment are critical for reducing the number of severe cases and deaths due to leptospirosis [9, 10]. Therefore, it is important to enhance the implementation of public health interventions in order to reduce the incidence of the disease.

In public health, decision-makers often have to allocate limited intervention resources in such a way as to slow down the outbreak of diseases and minimize their impacts (e.g. [11]). Knowledge about spatial patterns of diseases and their underlying processes have the potential to guide intervention efforts (e.g. [12]). In recent years, a growing number of studies using spatial analytical tools have been carried out, aiming at developing predictive maps of leptospirosis incidence to assist health authorities and policymakers to identify high-risk areas where prevention and surveillance measures should be strengthened (for a review, see [13]). Most of these studies have applied data-driven methods using leptospirosis notification data obtained from passive surveillance [13]. Data-driven methods require accurate and detailed epidemiological databases [14]. These methods should be applied to areas in which surveillance activities have effective coverage and the epidemiological database consists of a representative sample of the distribution of a disease. However, leptospirosis is often considered as an underreported and misdiagnosed disease even in endemic areas [3, 15]. The lack of data in many regions may make the estimation of its actual incidence and the identification of its determinants difficult to address adequately.

The assessment of the environmental and socioeconomic suitability (hereafter “suitability”) for human leptospirosis offers an alternative way to identify spatial patterns of the disease in areas where epidemiological data may be biased. While not a predictive approach, the suitability analysis can synthesize social and biophysical information to describe different conditions which may lead to the occurrence of the disease [16]. Spatial patterns of suitable conditions for the occurrence of infectious diseases have been previously assessed using a wide range of analytical tools (e.g. [17, 18]). Particularly, knowledge-based index and cluster analysis may provide complementary information about spatial patterns of the suitability for human leptospirosis. A knowledge-based index consists of the aggregation of a set of observable or hypothesized determinants of an event (in our case, the occurrence of leptospirosis) into a scalar variable by means of weighting criteria [19]. A knowledge-based index may be useful to rank sites according to the suitability for the occurrence of human leptospirosis. Since suitability cannot be observed directly, the main purpose of an index is to define suitability from variables that can be measured directly [19]. Consequently, it makes a theoretical concept operational since it aggregates real-world information into a format that is relevant and useful for decision-making [20]. However, similar levels of suitability may result from different environmental and socioeconomic conditions. Given that different combinations of determinants may imply distinct underlying processes for human leptospirosis, this information may be relevant for specific interventions planning. Cluster-based approach may provide insights about this topic. Cluster analysis has been previously used to identify homogeneous groupings or profiles in a wide variety of socio-ecological systems and these profiles have been considered as distinct socio-economic and environmental conditions in which different local mechanisms or processes may take place (e.g. [21]). In this sense, cluster analyses keep individual determinants discernable, as they are not merged into one final value, as typically occurs in the construction of an index. Cluster methods, however, do not automatically generate a profile hierarchy [22]. Hence, the knowledge-based index will reveal a suitability gradient for human leptospirosis while cluster analysis will contribute to identify distinct sets of determinants that may shape the suitability across a socio-ecological system [21, 22]. Finally, knowledge about the multiple determinants of the disease is required when both approaches are applied. There have been a large number of contributions regarding environmental and socio-economic determinants of human leptospirosis incidence around the world (e.g. [4, 5, 23]). Therefore, the application of both approaches to assess spatial patterns of suitability for human leptospirosis seems a feasible alternative to data-driven methods in areas where the representativeness of epidemiological databases is questionable.

The capital city of the province of Santa Fe (Argentina) is the core city of an urban agglomeration prone to suffer floods due to river overflow, heavy rains, or the combination of both. It exhibits an urban structure pattern typical of Latin American metropolises [24], with marginalized social groups located at the periphery and more affluent groups at the center of the largest cities. In addition, Ricardo et al. [25] found that there was a high proportion of people that inhabited riverside communities in the region with risky practices for leptospirosis and scarce knowledge about the disease. Finally, biases in national epidemiological databases have been previously reported [26, 27]. Therefore, our aims were twofold: (1) to identify the spatial distribution of the suitability for human leptospirosis and (2) to find distinct combinations of determinants that may lead to the occurrence of the disease across the urban agglomeration of Santa Fe.

## Material and methods

### Study area

**Fig. 1:**
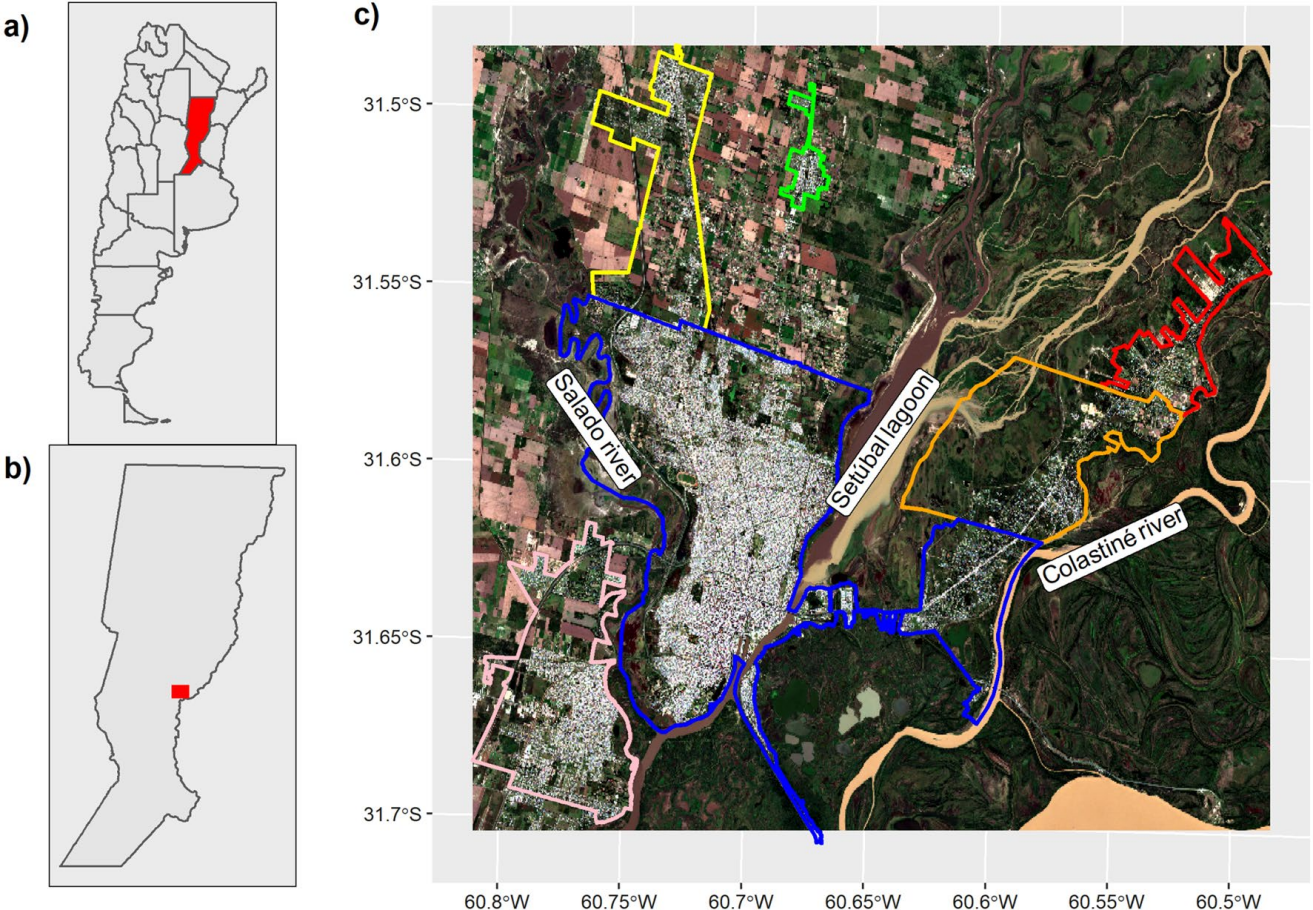
The urban agglomeration of Santa Fe. **A** Political boundaries of Argentina and its provinces are shown in black. The area of the province of Santa Fe is shown in red. **B** The political boundaries of the Santa Fe province are shown in black and the study area with a red squared. **C** The urban agglomeration of Santa Fe (UA of Santa Fe): the city of Santa Fe (blue boundaries), the city of Santo Tomé (pink), the city of San José del Rincón (orange), the city of Recreo (yellow), the township of Monte Vera (green), the township of Arroyo Leyes (red)

Our study area is composed of the following localities: the cities of Santa Fe, Santo Tomé, Recreo, San José del Rincón and two townships, Monte Vera and Arroyo Leyes (Fig. 1). We called our study area “the urban agglomeration of Santa Fe” (UA Santa Fe) [33]. The UA Santa Fe (31° 38ʹ 0ʺ S, 60° 42ʹ 0ʺ W) covers an approximate area of 705 sq. km and the population size is over 493,043 people [28]. The weather is temperate with a mean daily temperature of about 19.5 °C and approximate annual rainfall of 990.4 mm [29]. The topography is flat. The UA Santa Fe is crossed by the Salado River at the west and by the Paraná floodplain at the east (including the Colastiné River and Setúbal lagoon) (Fig. 1). The predominant vegetation types in the area are characterized by the confluence of the Paranaense (Interior Atlantic Forest) and Espinal phytogeographic provinces. The vegetation is strongly influenced by the floodplain of the Paraná River, which is composed of subtropical wet forest and gallery forest and different types of flooded savannahs and wetlands (rivers, streams, ponds and estuaries) [30, 31].

Santa Fe and Santo Tomé are the most densely built localities with the largest population size of the UA. In contrast, other UA localities present the highest rate of demographic growth and green spaces are composed of both planted and spontaneous vegetation [32]. The advantaged socioeconomic groups are mainly settled in downtown areas while the disadvantaged socioeconomic groups are mainly at the periphery of the cities of Santa Fe and Santo Tomé [33]. In the other localities, socioeconomic groups are not spatially segregated [33]. Therefore, all localities except the cities of Santa Fe and Santo Tomé were considered as suburban areas [34].

### Data source and collection

The epidemiological database was composed of both confirmed and probable human leptospirosis cases reported in the UA Santa Fe during the period 2010–2019 [35]. All records came from the National epidemiological surveillance system (SIVILA, Sistema de Vigilancia Laboratorial de Argentina). We geocoded the residential address of patients using the Google Geocoding API through Google Map. We aggregated probable and confirmed cases of human leptospirosis by census tracts and calculated the incidence of the disease based on the population reported for each census tract (Table 1). Environmental and socio-economic data were collected from different publicly available sources (Table 1). We calculated the Euclidean distance to open channels using the raster package [36] from R 2.8.1 version [37]. All variables were interpolated at a pixel size of 200 m since previous studies have found association patterns between human leptospirosis and socio-environmental determinants at similar spatial resolutions [38, 39].

**Table 1:** Data format, source and the interpolation method used for databases

Database	Data description	Source	Spatial interpolation
Demographic data^1^	Vector data. Census tracts represented by polygons	“Population” platform^4^	Area-weighting approach [114]
Socio-economic data^2^	Vector data. Census tracts represented by polygons	“Populations” platform^4^	Area-weighting approach [114]
Open channels	Vector data. Open channels represented by lines	Infrastructure of Spatial Data of the Province of Santa Fe website^5^	Euclidean distance
Elevation	Raster data. Digital Elevation Model (DEM) with 5 m resolution	National Geographic Institute website^6^	Minimum value of cells aggregated
Vegetation coverage	Raster data. Mean Normalized Difference Vegetation Index (NDVI) for the year 2018 with 5 m resolution	Sentinel 2 images	Bilinear method
Land cover types^3^	Raster data. Land cover raster for the year 2018 with 10 m resolution	Sentinel-1 and Sentinel-2 images	

References: (1) Demographic data: Number of inhabitants by census tract for the year 2010, (2) Socioeconomic data: Proportion of housings at the level of census tract: (a) without latrine, (b) without piped water supply, (c) without solid roof and/or with bare floor, (d) with the head of household with a high school diploma or above (tertiary and university degree) for the year 2010, (3) Land cover types: low vegetation, high vegetation, permanent and non-permanent water bodies and impervious surfaces, (4) https://poblaciones.org/, (5) https://www.santafe.gob.ar/idesf/portal, and (6) https://www.ign.gob.ar/

We rescaled variables using the formulas:

$Xir =\frac{Xi -\mathrm{min}\left(Xi\right)}{\mathrm{max}\left(Xi\right)-\mathrm{min}\left(Xi\right)}$, if the suitability increases with the variable values;

$Xir = 1 -\frac{Xi -\mathrm{min}\left(Xi\right)}{\mathrm{max}\left(Xi\right)-\mathrm{min}\left(Xi\right)}$, if the suitability decreases with the variable values.

*Xi* being the raw ith determinant and min and max being the minimum and the maximum functions, respectively. As a consequence, each rescaled determinant ranges from 0 (lowest suitability score) to 1 (highest suitability score).

#### Land cover classification

The land cover classification of UA Santa Fe was produced using Sentinel-1 RADAR satellite images and Sentinel-2 optical satellite images, freely provided by the Copernicus Program from the European Space Agency (ESA). 30 Sentinel-1 images at Ground Range Detected (GRD) level and 71 Sentinel-2 level 1A images, from January 2018 to December 2018, were downloaded on the ESA Copernicus Open Access Hub [40]. Sentinel-2 images were processed at level-2A using Sen2Cor from ESA to produce Normalized Difference Vegetation Index (NDVI) at each date.

Pre-processing of Sentinel-1 data was done using the Sentinel Application Platform (SNAP) developed by ESA following the usual steps to process Sentinel-1 GRD products: apply orbit file, subset to the study area, thermal noise removal, border noise removal, radiometric calibration, orthorectification and conversion to decibel (dB) [41]. Atmospheric corrections and cloud masking of Sentinel-2 data were performed using the MAJA processor developed by CNES, CESBIO and DLR [42, 43]. A pixel-based classification using S1 and S2 time-series was then performed to produce a land cover map of five classes: low vegetation, high vegetation, permanent and non-permanent water bodies and impervious surfaces [44–46] (Table 1).

Based on the land cover classification previously created, we used the raster [36] and landscape metrics [47] packages from R [37] to generate the following environmental and landscape heterogeneity determinants of human leptospirosis at a pixel size of 200 m: the distance to waterbodies, the proportion of waterbodies, high vegetation, low vegetation and impervious (built-up) surface, the number of patches, the edge density and the Shannon diversity (Table 1).

### The conceptual framework

We considered two scenarios: a Conservative one (CSc), which considers only determinants whose association with leptospirosis is widely supported by the literature and expert knowledge, and an Explorative one (ESc) which also includes determinants that have rarely been investigated. Therefore, our conceptual framework was based on the following assumptions:

#### The Conservative scenario (CSc)

Precarious living conditions such as lack of adequate housing, clothing, food and basic services (piped water, sewage and garbage collection) promote environmental contamination, the thriving of rodents, and exposure to the bacteria [39, 48]. On the other hand, education enhances the likelihood of using preventive practices [25, 39]. Therefore, the higher the level of precarious living conditions and the lower the access to education, the greater the suitability for human leptospirosis.

Leptospirosis infection risk is inversely associated with terrain elevation given that the contact with water and humid soil is more likely in lower terrains [39, 49]. Consequently, the lower the elevation, the higher the suitability for human leptospirosis.

Human leptospirosis incidence is generally associated to areas with abundant water bodies, such as lakes, or in proximity to a river [50, 51]. Studies have reported that the presence of a river adjacent to human settlement increased the risk of leptospirosis [52, 53]. The nearer to and the greater proportion of water bodies, the higher the suitability for human leptospirosis. On the other hand, the proximity of households to open drainage systems and direct contact with sewage, flooding water and runoff have been associated with increased risk of infection [54–58]. The nearer to open urban channels, the higher the suitability for the disease.

#### The Explorative scenario (ESc)

The abundance of many rodent species is positively influenced by vegetation cover as it provides food and shelter to these animals [59, 60]. High density of rodents could boost leptospires shedding into the environment as well as increase the risk of transmission among reservoir animals [61]. On the other hand, greater vegetation cover is associated with increased humidity, lower ambient temperatures and solar radiation, all of which can enhance the persistence of free-living stages of *Leptospira* spp. [62–64]. Therefore, the association between the suitability for human leptospirosis and vegetation coverage was considered positive.

In urban environments, infected synanthropic rodents will be found in areas covered by a mix of buildings and spontaneous and/or cultivated green spaces, increasing the transmission of *Leptospira* spp. Additionally, urban green spaces will provide shaded areas, puddles, moist soils, and lower temperatures than those sectors where pavement and buildings predominate. We assumed that the suitability for human leptospirosis is positively associated to landscape heterogeneity and negatively associated to built-up areas.

In urban environments, infected animals were mainly found in areas covered by a mix of buildings and spontaneous and/or cultivated green spaces [65, 66]. The dominance of synanthropic rodents and the relatively high abundance that they can reach in those heterogeneous areas is likely to result in greater transmission of *Leptospira* spp. [66]. Increasing landscape heterogeneity in human settlements may favor leptospiral infection in synanthropic rodent species [65, 66]. On the other hand, agricultural areas may be suitable environments for the bacteria because of greater vegetation cover, humidity and wet soils [62, 64, 67]. Instead, urban environments may be heterogeneous in terms of environmental suitability since leptospiral survival would be favored in green spaces that provide shaded areas, puddles, moist soils, and lower temperatures than those sectors where pavement and buildings predominate [62, 68–71]. Therefore, the association between human leptospirosis and landscape heterogeneity determinants was considered positive. In contrast, the association between the suitability and the percentage coverage of built-up areas was considered negative.

### Assessment of multi-collinearity

In order to assess information redundancy in the dataset, we used the Variance Inflation Factor (VIF) and Pearson correlation coefficient (r) between determinants [72]. All determinants with a VIF < 5 and a r < 0.8 were considered for further analyses. We used a stepwise procedure: we calculated VIF for each determinant and excluded the one with the highest VIF (greater than the threshold). Then, we repeated the procedure until no determinant with VIF > 5 remained [73]. Finally, we calculated the pairwise Pearson coefficient to check if all the remaining determinants were not correlated.

### Knowledge-based index

To combine human leptospirosis determinant layers based on the spatial distribution of the suitability defined above we used the Zonation algorithm [74]. This algorithm takes into account the spatial distribution of features in the landscape associated with the occurrence of leptospirosis to determine the priority value of an area. It starts from the full landscape, and then iteratively discards locations (grid cells) of lowest priority value from the edge of the remaining area, thus maintaining a high degree of structural connectivity [74]. Consequently, this allows the identification of a nested sequence of aggregated landscape structures with locations of highest priority value remaining until the last iteration. In health care systems, nested zoning may be an interesting approach for guiding geographically-prioritized limited resource allocation during the decision-making process [75].

One of the approaches that Zonation uses for defining the importance of locations is the benefit function [76]. In the additive benefit function the value of a priority area is given by the sum over feature-specific values of representation in the landscape [76]. Since we assumed that the most suitable areas are those where multiple determinants for the occurrence of human leptospirosis presented the highest values [4], we used Zonation’s additive benefit function to generate a suitability gradient ranging from 0 to 1 [76]. The lowest suitability score does not mean protective socio-environmental conditions since low values may be caused by features whose highest values occur in feature-poor regions (cells with the lowest values for many features in them) [76].

Feature weighting allows Zonation to maintain a balance among features in the outcomes of the analyses. In CSc all determinants were assumed to have equal importance (i.e., weight *W*_*i*_ = 1 for each determinant *X*_*i*_^*r*^) given the lack of information on the relative importance of determinants [77]. In the ESc, we considered a greater number of environmental determinants than socio-economic ones. The group with the largest number of determinants may have the greatest influence on Zonation outcomes. In order to avoid unequal aggregate weights based on different number of determinants within each group, we assigned the same aggregate weight (*W*_*i*_ = 6) to each group of determinants and rescaled the weights of determinants within each group to sum up to the aggregate weight [78].

### Cluster analysis

We identified areas with distinct socio-environmental characteristics (here referred to as ‘suitability profiles’) across the UA Santa Fe by means of the following steps. We first applied a principal component analysis (PCA) on the determinants for both the conservative and explorative scenarios [79]. We considered the first five principal components (PCs) which represented about 85% of the cumulated data variance. Thus, we removed random fluctuations which generally constitute the bulk of the variance retained in the last axes (non-systematic variations contained in the data) [80]. This improves clustering by producing more homogeneous classes [80]. We then used hierarchical and partitioning (k-means) algorithms to the PCs [79]. Hierarchical clustering assigns sites into groups based on the similarity between them using Ward’s minimum variance criterion to minimize the total within-cluster variance. However, the partition obtained is not always optimal because of the structure of nested partitions in the obtained dendrogram. On the other hand, while the k-means algorithm efficiently partitions the data into k groups, its outcome is sensitive to initialization. Therefore, we used the hierarchical algorithm (Ward’s method) to define the initial conditions (i.e., the cluster barycenter) for the k-means [81]. We performed cluster analysis using the databases of both scenarios. We set the optimal number of clusters to three in both scenarios, as it was estimated by most of the 30 indices listed in Charrad et al. [82].

### Comparison between both scenarios: CSc and ESc

To compare the suitability gradients from the knowledge-based index, we used the Pearson correlation coefficient and the Fuzzy Inference System (FIS) [83]. The evaluation of the spatial similarities between both scenarios with the Pearson correlation coefficient is based on a cell-by-cell comparison. However, a cell-by-cell comparison may register a disagreement between cells even when the overall spatial patterns are essentially the same [83]. The FIS comparison algorithm offers an alternative approach. It compares the characteristics of polygons rather than cells found in both maps [83]. The characteristics that are taken into account in this evaluation are area of intersection, area of disagreement and size of polygon [83]. We reclassified suitability gradients into 5 bins (0, 0.25, 0.5, 0.75, 0.9, 1) and implemented the FIS comparison algorithm available at Map Comparison Kit [83]. The value for similarity ranges from 0 to 1, with 0 corresponding to two completely dissimilar maps and 1 to maps with completely matching cells. We considered a similarity threshold of 0.55 [84]. We applied a correspondence analysis to compare the suitability profiles obtained from the cluster analysis.

### Comparison between the spatial distribution of the suitability for human leptospirosis and the distribution of incidence of the disease

We assessed the level of agreement between the spatial distribution of the suitability for human leptospirosis and the distribution of the incidence of the disease in the UA of Santa Fe given that it can be considered as an indicative measure of the strength by which environmental and socioeconomic conditions influence the occurrence of the disease [18, 85]. We applied the Pearson correlation coefficient to compare suitability scores from the knowledge-based index and the incidence of the disease. We compared the distribution of leptospirosis incidence between clusters using non-parametric Kruskal–Wallis and Wilcoxon tests. All analyses were performed using the free Statistical software R 2.8.1 version [37].

## Results

### Epidemiological and socio-environmental databases

In the UA Santa Fe, a total of 291 human leptospirosis cases were reported for the period 2010–2019 by the SIVILA: 92 confirmed cases and 199 probable cases (31.6% and 68.4%, respectively). Most records came from the city of Santa Fe (233 records, 80.1%). Based on the quality of locality descriptions, we were able to geocode 164 records (56.36%): 44 confirmed cases and 120 probable cases.

We removed the proportion of housings with bare floor from both, CSc and ESc scenarios, and proportion of low vegetation, NDVI and edge density from ESc scenario because of a VIF > 5 (Table 2).

**Table 2:** Variance Inflation Factor (VIF) and Pearson coefficient (R) for human leptospirosis determinants in both the Conservative and Explorative scenarios (Csc and Esc, respectively)

Determinants	Csc		Esc	
	VIF	R	VIF	R
Indoor water supply*	1.46	0.43	2.27	− 0.66
Solid roof*	3.01	− 0.74	3.56	− 0.75
Latrine*	2.69	− 0.73	2.84	− 0.75
High school or more^+^	3.74	− 0.74	3.94	− 0.75
Elevation	1.68	− 0.49	1.7	− 0.47
Distance to channel	1.14	0.26	1.15	0.27
Distance to water bodies	1.45	0.47	1.47	0.45
Proportion of water bodies	1.4	− 0.49	1.81	− 0.45
Proportion of high vegetation	–	–	1.91	− 0.39
Proportion of built-up surface	–	–	3.1	− 0.66
Number of patches	–	–	2.32	0.63
Shannon diversity	–	–	2.64	0.63

^*^Proportion of housings without indoor water supply, solid roof or latrine

^+^Proportion of housings with the head of household with a high school education or more

### Knowledge-based index

**Fig. 2:**
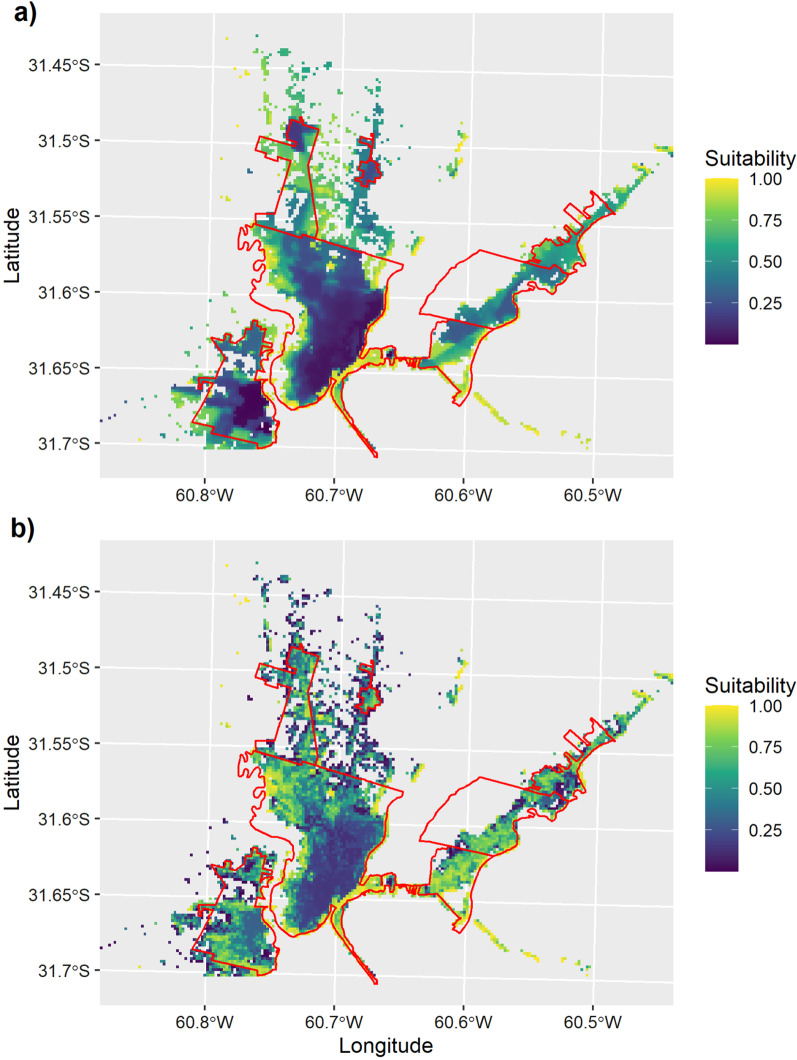
The gradient of the environmental and socioeconomic suitability (hereafter “suitability”) for human leptospirosis across the urban agglomeration of Santa Fe. **A** The suitability for human leptospirosis according to the Conservative scenario. **B** The suitability for the human leptospirosis according to the Explorative scenario

**Fig. 3:**
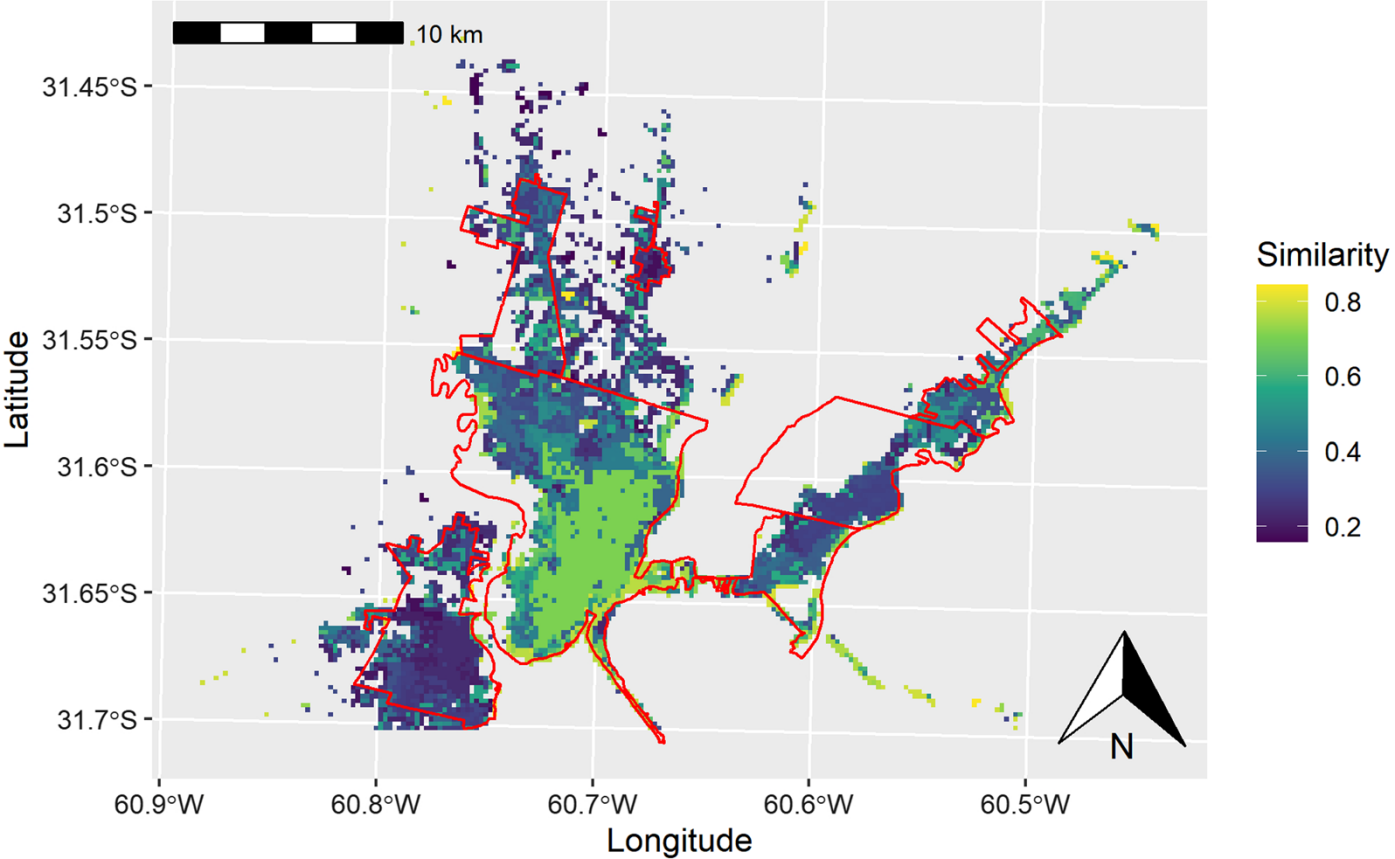
The Fuzzy Inference System comparison between the environmental and socio-economic suitability for human leptospirosis obtained from both the Conservative and Explorative scenarios

We found an increasing suitability gradient for the occurrence of human leptospirosis from downtown areas of the cities of Santa Fe and Santo Tomé towards peri-urban and suburban areas (Fig. 2). Suitability gradients obtained from both scenarios, CSc and ESc, were positively correlated (*r* = 0.55, *P*-value < 0.001). Additionally, we obtained a low degree of similarity between both scenarios according to the Fuzzy Inference System (Fuzzy global matching = 0.44). Greater differences in suitability scores between both scenarios were observed at the city of Santo Tomé and suburban areas of the UA (Fig. 3).

### Cluster analysis

**Fig. 4:**
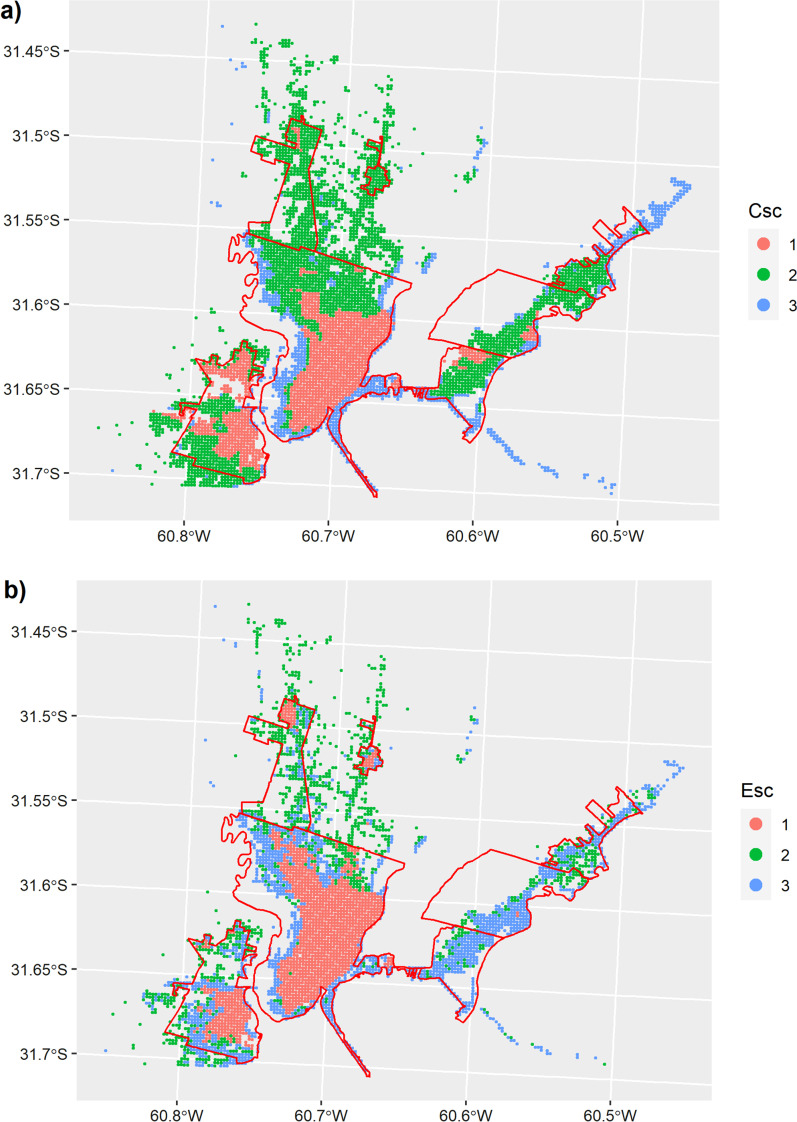
Aggregation of sites according to environmental and socio-economic determinants for human leptospirosis obtained from the cluster analysis and both the Conservative (**a**) and Explorative (**b**) scenarios

**Fig. 5:**
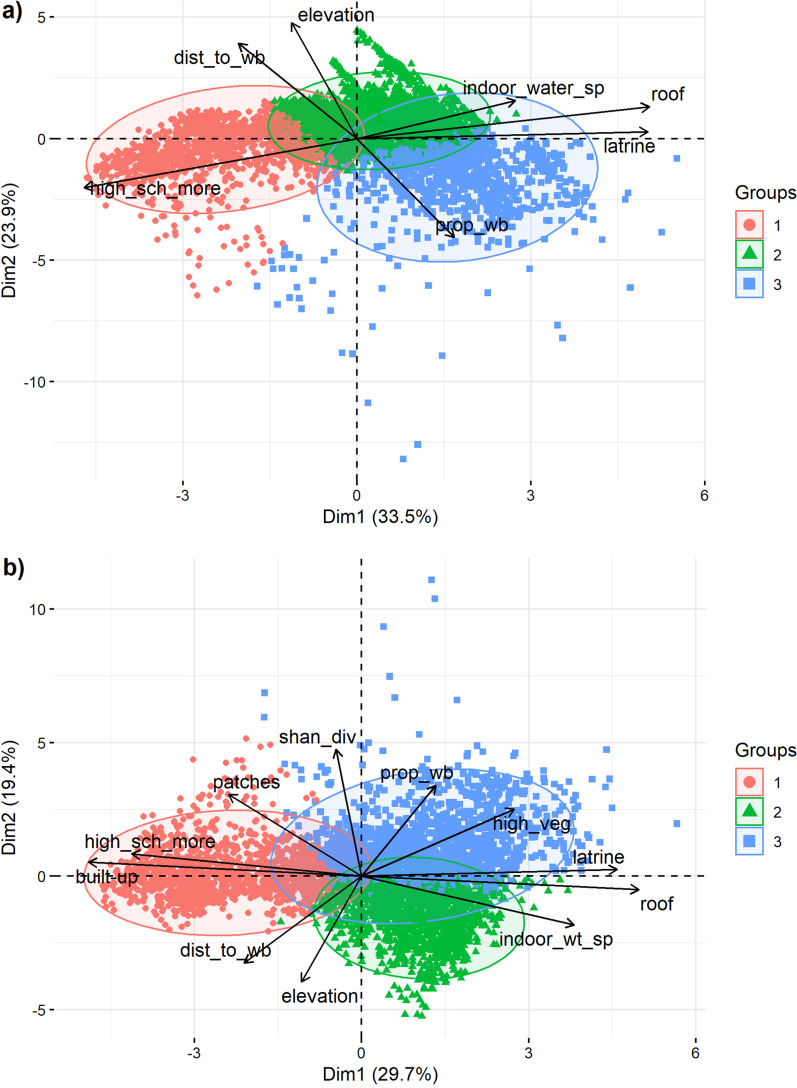
Biplot of environmental and socioeconomic determinants of human leptospirosis and sites of the Urban Agglomaration of Santa Fe (UA of Santa Fe) based on a Principal Component Analysis. Sites of the UA of Santa Fe are represented by points. Determinants for human leptospirosis are represented by arrows. Arrow orientation represent the direction of the steepest increase of the determinant. Arrow length indicates the relative importance of determinants in the model, the angle between arrows and axes indicates the degree of correlation between them. Socioeconomic determinants: Proportion of housings without indoor water supply (“indoor_water_sp”), solid roof (“roof”), latrine (“latrine”), and with the head of household with a high school education or more (“high_sch_more”). Environmental determinants: elevation (“elevation”), distance to channel (“dist_to_channel”), water bodies (“dist_to_wb”), proportion of water bodies (“prop_wb”), high vegetation (“high_veg”), impervious (built-up) surface, number of patches (“patches”) and Shannon diversity (“shan_div”)

**Fig. 6:**
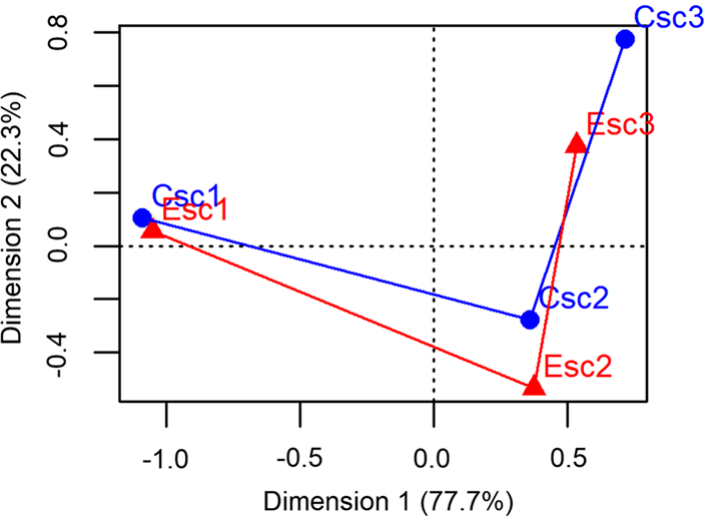
Correspondence biplot of clusters (“suitability profiles”) from both the Conservative and Explorative scenarios for the Urban Agglomeration of Santa Fe. Blue circles correspond to clusters from the Conservative scenario (Csc). Red triangles represent clusters from the Explorative scenario (Esc). Clusters of each scenario are differentiated with a number added as a suffix (1, 2 and 3)

We did not find significant differences between socio-environmental profiles from both scenarios. The cluster 1 (CSc1 and ESc1) mainly included downtown areas of Santa Fe and Santo Tomé, while cluster 2 (CSc2 and ESc2) and cluster 3 (CSc3 and ESc3) overlapped peripheral and suburban areas of the UA Santa Fe (Fig. 4). In the CSc, cluster 2 mainly overlapped suburban areas while cluster 3, peripheral areas of the Santa Fe city (Fig. 4a). In contrast, cluster 3 included suburban areas in the ESc (Fig. 4b). Clusters were distributed along the first and second PC (Fig. 5). According to the first PC, cluster 1 (CSc1 and ESc1) was characterized by higher levels of education and better housing conditions than cluster 2 (CSc2 and ESc2) and cluster 3 (CSc3 and ESc3) (Fig. 5). According to the second PC, cluster 2 (CSc2 and ESc2) and cluster 3 (CSc3 and ESc3) presented different environmental conditions suitable for human leptospirosis (Fig. 5). Differences in the geographic space of clusters between both scenarios were mainly presented in cluster 2 and cluster 3 (Fig. 6).

### Comparison between the spatial distribution of the suitability for human leptospirosis and the distribution of incidence of the disease

We obtained the highest incidence rates in suburban and peri-urban areas where environmental and socioeconomic suitable conditions for human leptospirosis predominate. Knowledge-based index predictions for both scenarios returned significant (*P* < 0.001) positive Pearson correlation coefficients when considering all incidence (CSc *r* = 0.21, *P* < 0.001 and ESc *r* = 0.19, *P* < 0.001) and non-null incidence values (CSc *r* = 0.35, *P* < 0.001 and ESc *r* = 0.26, *P* < 0.001) (Additional file 1a–d). The highest incidence rates of leptospirosis cases overlapped CSc2 and CSc3 (Wilcoxon test: CSc1–CSc2, *P* < 0.001; CSc1–CSc3, *P* < 0.001; CSc2–CSc3, *P* = 0.7415) (Additional file 1e) and ESc3 (Wilcoxon test: ESc1–ESc2, *P* = 0.69; ESc1–ESc3, *P* < 0.001; ESc2–ESc3, *P* < 0.001) (Additional file 1f). We obtained high overlapping between the distributions of incidence in all clusters (> 50%) for both scenarios and consequently, statistical differences are due to the presence of outliers (Additional file 1e, f).

## Discussion

We used a comprehensive approach based on reliable bibliographic information and expert knowledge in order to contribute to the territorial representation of the suitability for human leptospirosis in an urban agglomeration where the representativeness of epidemiological databases was questionable. Our results indicated that the suitability for the disease is spatially heterogeneous in the UA of Santa Fe, being more suitable towards suburban areas of the urban agglomeration. The identification of distinct profiles through the cluster analysis helped to understand this spatial heterogeneity in the suitability since it provided a different set of possible drivers for the occurrence of the disease across the UA Santa Fe. The usage of different scenarios based on different assumptions about leptospirosis determinants did not lead to significant differences in the spatial arrangement of the suitability for the disease. In contrast, we observed some spatial mismatches in peripheral and suburban areas. Therefore, the methodology is a useful tool for the spatial representation of the suitability from human leptospirosis determinants widely supported by the literature and can be thought as an evolutive and perfectible scheme as more studies are performed in the area and novel information regarding these or other determinants become available. Our approach can be a valuable tool for decision-makers since it can serve as a baseline to plan preventive measures and to monitor human leptospirosis determinants.

Knowledge about association patterns between the occurrence of infectious diseases and environmental and socio-economic drivers enables the construction of suitability gradients for the occurrence of those diseases [13]. Our knowledge-based index identified high levels of suitability for human leptospirosis in peripheral areas of Santa Fe and Santo Tomé and suburban areas of the UA. This spatial pattern agrees with the Socio-economic Residential Segregation reported in previous studies for the region [33]. The downtown areas and their surroundings in the cities of Santo Tomé and Santa Fe are equipped with better infrastructure and inhabited by advantaged socioeconomic groups while peripheral areas of these cities and suburban areas of the UA include self-construction or informal and state planned neighborhoods with few or without public services, mainly inhabited by disadvantaged socioeconomic groups [33]. This spatial pattern of suitability may have derived from various economic, social, and demographic processes. Two main urbanization processes can be recognized in the UA Santa Fe [86]: middle income settlements close to industries and the center of the cities of Santa Fe and Santo Tomé during a stage of industrial development (mainly during the 1950s–1970s); and informal impoverished settlements in low topography areas (beginning in the 1970s–1980s) and nowadays exposed to flooding and close to dumps and landfills. As social and spatial inequalities in housing, health, education, or financial resources were settled, new ecological niches for leptospirosis may have emerged [87, 88]. Additionally, the highest population growth rates were reported at the suburb of the UA Santa Fe in the last two censuses (2001–2010) [24, 32]. The inadequate land use planning policies and infrastructure provision in rapidly growing and expanding settlements could create the conditions for future outbreaks of leptospirosis [87, 89]. Therefore, knowledge about social processes that shape these spatial patterns may help to understand, predict, prevent and control the emergence of suitable conditions for human leptospirosis.

Leptospirosis is commonly reported in urban settings where both socioeconomic and environmental suitable conditions coexist (e.g. [39]). We identified two profiles of suitability (cluster 2 and 3) characterized by both environmental and socioeconomic determinants that overlapped areas with the highest suitability scores according to the knowledge-based index. Differences between these profiles were based mainly on environmental determinants. However, this result should be interpreted with caution as two limitations have to be considered. First, although CSc2 and ESc2 included sites with a lower proportion and greater distance to water bodies and a higher elevation than CSc3 and ESc3, these sites are still prone to being flooded mainly during extraordinarily large floods of the Salado and Paraná rivers (see [90, 91]). Even the center of Santa Fe is prone to being flooded during the largest overflows of the rivers [90]. Despite of environmental differences reported in our results, the incidence of human leptospirosis may rise and be more widespread after a flood event involving areas that are at higher elevation and far away from the water bodies [92, 93]. Second, we could not find differences based on socioeconomic determinants, but different socioeconomic groups can still be found. CSc2 and ESc2 encompassed small farms and horticultural lands settled in peripheral areas of Santo Tomé and Santa Fe [91, 94]. In contrast, CSc3 and ESc3 encompassed riverside communities settled mainly in the periphery of Santa Fe and suburban areas in the east. These riverside communities are prone to being flooded and characterized by precarious houses intermixed with patches of spontaneous vegetation and small dump sites [25] (*pers. obs.*). Many of the residents of these communities work in the informal market as subsistence fishermen, hunters or farmers and have several domestic animals non vaccinated against leptospirosis [25] (*pers. obs.*). Therefore, the heterogeneity of these suitable conditions for human leptospirosis found in suburban areas should be taken into account in order to guide prevention and control actions.

Although the two scenarios evaluated did not differ significantly in the spatial arrangement of the suitability for the disease, we found some spatial mismatches at intermediate levels of suitability. For instance, CSc1 overlapped suburban areas at the east characterized by the presence of first and second households belonging to groups with medium–high income [32]. In contrast, these areas were assigned to clusters ESc2 and ESc3 when environmental and landscape heterogeneity determinants were considered in the ESc. These areas are characterized by a lower density of human settlements which are surrounded by patches of introduced and spontaneous vegetation, water bodies and unpaved streets [24]. In addition, middle-high income groups often settle in suburban areas to enjoy nature and practice outdoor activities such as water sports, fishing among others [32, 95]. These practices along with environmental conditions may increase the probability of exposure to pathogenic leptospires [6]. Therefore, despite of the presence of advantaged socioeconomic groups, suitable conditions for human leptospirosis may still occur.

The low degree of agreement between the suitability scores and suitability profiles for human leptospirosis and the incidence of the disease in the UA Santa Fe for both scenarios was expected for the following reasons:

1. Suitable conditions do not necessarily imply the occurrence of human leptospirosis
  Estimates of suitability were based only on environmental and socioeconomic drivers while other factors may influence the incidence of the disease [96]. Two previous studies conducted in Santa Fe and suburban areas at the east of the UA, Vanasco et al. [97] and Ricardo et al. [98], found high prevalence of anti-Leptospira antibodies in rodent species at sites with different environmental and socioeconomic settings. In our suitability gradient for human leptospirosis, even the lowest suitability scores overlapped areas where high seroprevalence was found in rodents. In this sense, rodent species may contribute to the circulation of the bacteria across the UA Santa Fe, but it may not be enough to shape the occurrence of human infections [25]. Future studies are necessary to assess this hypothesis in the region. On the other hand, chemoprophylaxis campaigns are commonly used to prevent leptospirosis outbreaks in populations affected by floods in endemic areas [9]. According to López et al. [92], a stronger preventive campaign, including chemoprophylaxis, may explain the lower incidence of leptospirosis observed during the flood of 2015–2016 with respect to the flood of 2009–2010 that affected the region. This campaign not only may have led to an attenuation of the outbreak but also may explain changes in spatial patterns of the disease [92].
2. Inherent problems in the epidemiological data collection methods
  The national epidemiological database lacks relevant information about the disease. First, information about transmission pathways is lacking. Thus, we could not differentiate whether leptospirosis cases were due to direct contact with animals or environmental exposure. However, biases based on transmission pathways may be negligible since in Argentina, the main risk factor for leptospirosis is persistent contact with flooded environments [26]. Second, it is possible that locations where infection took place are different from where the disease case was reported. In the national epidemiological database, most of human leptospirosis records that include information on the patient’s home address do not have information on whether the patient indicates a different locality for where exposure may have occurred. This is particularly a problem for analyses performed with a spatial resolution that includes housings and its surroundings such as the one conducted in the present study [96]. Third, the low level of completeness in the locality description is also problematic. We were unable to assign geographic coordinates to almost half of the cases since many locality descriptions included only the name of the city or the type of environment (rural or urban). Depending on the spatial scale considered, this missing information could greatly affect the performance of the analyses. The current level of completeness of the database was sufficient for exploratory analyses conducted previously in the region that provided some insights on association patterns between the dynamics of human leptospirosis and socioeconomic and environmental drivers at larger spatial scales [26, 92]. However, greater data quality would enable more in-depth studies that can provide better descriptions of the risk groups or areas to help guide allocation of health resources [99]. In Argentina, the surveillance system was modified in 2019, linking the patient health data to the National Registry of Persons (Renaper) database, thereby improving the completeness of the database.
3. The weighting criteria applied to the leptospirosis determinants
  Finally, differences between leptospirosis incidence and the spatial distribution of the suitability may be due to the weighting criteria applied for leptospirosis determinants. The choice of the aggregation method influences the results, making the aggregation criteria a subjective decision in both the index construction and clustering process. Different weighting criteria have been proposed to avoid biases due to the choice of aggregation criteria used [100, 101]. Therefore, future work could explore the outcomes obtained by applying different weighting criteria. However, we think that the spatial distribution of the suitability for human leptospirosis presented here is robust based on our careful and thorough determinant selection procedure, on the usage of different scenarios and on the clustering approach employed in this study.

Strengthening the health system in order to enhance its capacity regarding prevention, surveillance and control actions has been proved to be useful to reduce the frequency of epidemics [102]. However, resources are often limited and consequently, resource allocation is a central part of the decision-making process in health care systems [75]. Our findings may contribute to allocate limited funds available for health system since the spatial distribution of the suitability for human leptospirosis was obtained. By means of a nested ranking approach, we identified areas where greater efforts would be required to reduce the occurrence of leptospirosis outbreaks given that most determinants supported by the scientific literature coexist.

Some considerations must be taken into account to improve the spatial arrangement of the obtained suitability:

(i) Additional environmental and socioeconomic determinants supported by the literature could be explored, such as the presence of formal and informal garbage dumps, the proportion of empty properties, the proportion of the population engaged in freshwater fishing, collecting and processing solid waste, horticulture, among others [4, 5]. However, this information is often not available, mainly in the developing world, as it is the case for our study area. Thus, the consideration of additional socio-environmental determinants would require conducting additional fieldwork to collect relevant data.
(ii) Suitability scores may vary over time given that variable spatio-temporal patterns of human leptospirosis incidence have been widely reported (e.g. [103, 104]). In Argentina, the highest incidence rates of human leptospirosis were recorded in seasons with warm and moderate temperatures (summer and autumn) [26, 92, 105]. These outbreaks occurred during the weeks of heavy rainfall or 1 to 2 weeks after the onset of such climatic conditions or following floods [92]. Moreover, many mammal species can act as reservoirs for *Leptospira* spp. [106]. In addition, infection prevalence by *Leptospira* spp. has been found to be higher in wildlife occupying urban habitats than natural environments, and this trend appears to be particularly significant for rodents [106]. In the region, several rodent species such as *Rattus norvegicus*, *R. rattus* and *Mus musculus* have been recorded in urban environments [60, 97]. This can lead to regular human exposure to these species and their excreta. Despite risks posed by urban rodent infestation, the distribution, prevalence, diversity and dynamics of *Leptospira* spp. infections in urban rodent populations remains largely unknown, affecting the ability of local authorities to develop effective intervention strategies. Therefore, temporal variations in the spatial configuration of water bodies, rates of rainfall, the spatial distribution of animal reservoirs and rodent activity could be assessed as human leptospirosis determinants to evaluate changes in the spatial arrangement of the suitability across the time [92, 103, 107].
(iii) Human groups have often promoted or limited the spread of infectious diseases through culturally coded patterns of behavior, modes of production and changing social relationships which led to changes in relationships among infectious disease agents, their human and animal hosts, and the environment in which the interaction takes place [108]. As we stated above, the observed suitability gradient may have been the result of socio-demographic, cultural and economic processes that occurred in the UA Santa Fe over time, mainly during the last decades [32, 86]. Additionally, the higher growth rate in suburban localities with respect to the largest cities of the UA Santa Fe may lead to changes in the current urban structure and distribution of the population [32], and probably, on the spatial distribution of the suitability for the disease. Future studies should analyze the effect of the economic, social and political dynamics on the occurrence of human leptospirosis in the region in order to understand, prevent or control the ultimate causes of the disease [109].
(iv) The Modifiable Areal Unit Problem (MAUP) should be explored whenever possible. The MAUP is composed of two separate but closely related problems. First, the spatial scale determines the range of patterns and processes that can be detected on a landscape, and consequently researchers must be aware of the uncertainties associated with changes in spatial scales (“the scale problem”) [110]. In this study we described suitable conditions for the human leptospirosis in terms of environmental and socioeconomic determinants at a neighborhood-level (housings and their surroundings). We considered this to be an appropriate spatial scale since previous studies found significant associations between incidence of the disease and its determinants at similar scales [38, 39, 111]. However, since spatial patterns of the disease are scale dependent (e.g. [111]), future studies should consider different spatial scales to obtain a more exhaustive description of suitable conditions for the occurrence of human leptospirosis in the UA Santa Fe. The second one, the aggregation problem, refers to variation in spatial pattern and processes due to the use of alternative combinations of areal units at equal or similar scales [110]. Socio-economic variables are often obtained from census data that are aggregated over arbitrary areal units. In such circumstances, researchers have no control over how variable aggregations are made and/or how those areal units are determined [112]. If the results are sensitive to change in boundaries, caution should be exercised when interpreting apparent associations between environmental exposures and health effects [113]. Although we cannot disentangle the spatial distribution of socioeconomic databases from that associated with arbitrary aggregations, we think that these databases are still useful according to our objectives. In previous studies, spatial patterns of processes such as social vulnerability, vulnerability to natural disasters, quality of life, accessibility to green spaces, among others, were analyzed using the same source of socioeconomic data that we applied in this work [114–117].
(v) During the construction process of an index and clustering, uncertainty and sensitivity analysis should be also performed whenever possible [118, 119]. Uncertainty analysis is performed to investigate variations in the index and clustering outputs that are generated from uncertainty in parameter inputs [118]. Sensitivity analysis follows uncertainty analysis as it assesses how variations in the index and clustering outputs can be apportioned, qualitatively or quantitatively, to different input sources [118]. Therefore, uncertainty and sensitivity analyses offer a way to assess the adequacy of index and clustering and establish what factors affect their outputs [120]. Uncertainty will arise from all of the arbitrary choices performed in each step during the construction process of an index and clustering. These choices are mainly made based on both statistical procedure, such as the standardization of data, and alternative epidemiological hypothesis [118]. Regarding statistical procedures, researchers should compare our approach with alternative statistical procedure in order to assess their impact on the spatial patterns of the disease (e.g. [118]). On the other hand, uncertainty will also arise whenever alternative hypotheses emerge. For instance, one of the most important sources of uncertainty is the selection of the determinants that are aggregated according to competing hypothesis (e.g. [121]). In our study, the similarity of knowledge-based index and clustering outputs between the Conservative and Explorative scenarios indicated minor effects of additional human leptospirosis determinants to those widely supported by the scientific literature. This suggest that variables in the Conservative scenario may be among the most important determinants of the spatial arrangement of the suitability of human leptospirosis in the UA of Santa Fe. We expect new findings may still arise when comparing alternative models that combine and integrate the determinants of the disease considered here and others in different ways. Therefore, we consider our approach as an informative tool for an initial assessment of spatial patterns of the human leptospirosis in the UA of Santa Fe that can be updated as novel information regarding the underlying processes of the disease become available. However, sensitivity analyses are still lacking and assessing and comparing the effect of different sources of uncertainty based on alternative hypothesis and not only statistical procedure will also shed light on the knowledge of the disease, and consequently, may impact on the spatial arrangement of the suitability of the disease in the region.

Finally, it is important to note that suitability maps help to decide where to act but do not provide insights on the specific interventions needed there. A multi-sectorial and multi-stakeholder exchange is required to set priorities among prevention, surveillance and control measures and allocate resources across the region to reduce disease incidence and improve response capacity to leptospirosis outbreaks [122, 123]. It is crucial that the communities are engaged in a participatory manner and are supported to undertake healthy village initiatives, in ways that respect cultural values, traditions, and local governance structures [124].

## Conclusions

We presented a method to analyze the spatial heterogeneity of the suitability for the occurrence of human leptospirosis that is particularly useful in areas where high-quality epidemiological data are lacking. Our approach can be more broadly used to explore the spatial distribution of the suitability for the occurrence of infectious diseases caused by parasites that have a free-living stage. The spatial distribution of the suitability obtained in this study is not intended to be interpreted as definitive, instead, they should be considered as estimates based on the available evidence, and the scientists’ interpretation of that evidence. Although we used environmental and socio-economic determinants widely supported by the literature and expert knowledge, using this approach with alternative determinants would provide further insights. Using this novel and integrative approach and the available information for the UA Santa Fe, we obtained a suitability map for the occurrence of human leptospirosis with clear and robust patterns. The identification of a limited number of distinct suitability profiles by the cluster analysis complemented our knowledge-based index approach and enabled us to distinguish potential underlying processes that shape the suitability for the disease. As the current spatial distribution of the suitability may have been shaped by social processes that took place in the past, the recent higher growth rate in suburban areas of the urban agglomeration may change the underlying processes that shape the suitability for leptospiral exposure. Therefore, our approach may contribute to analyze these changes in the future. Our methodology can be extended with the use of alternative scenarios or projected data (e.g., land use change or population density projections) to better understand potential changes in the spatial distribution of the suitability for the disease. The resulting insights suggest that prevention strategies efforts should be spatially heterogeneous across the UA Santa Fe. Our results may help to prioritize areas and social groups and hence, guide the allocation of limited health resources more appropriately. This is an important step towards developing methods that can help to reduce the incidence of the disease mainly in developing countries that are the most affected by the burden of leptospirosis and that generally lack high-quality surveillance data.

## Supplementary Information

**Additional file 1.** Comparisons between human leptospirosis incidence rate in the Urban agglomeration of Santa Fe and the environmental and socio-economic suitability (“suitability”) and suitability profiles (“clusters”) for the disease. References: Pearson correlation between the suitability for the disease and human leptospirosis incidence rate using all incidence data for the Conservative scenario (a) and the Explorative scenario (b). Pearson correlation between the suitability for the disease and human leptospirosis incidence rate using non-null incidence data for the Conservative scenario (c) and the Explorative scenario (d). Boxplot of human leptospirosis incidence rate in the clusters for the Conservative scenario (e) and the Explorative scenario (f).

## Data Availability

The datasets used and/or analysed during the current study are available from the corresponding author on reasonable request.

## Abbreviations

Suitability: The environmental and socioeconomic suitability
UA Santa Fe: The urban agglomeration of Santa Fe
