# Supplementary figures and images for: Determining the spatial distribution of environmental and socio-economic suitability for human leptospirosis in the face of limited epidemiological data
Source: Infect Dis Poverty. 2022 Aug 4;11:86. doi: 10.1186/s40249-022-01010-x (PMC9351081; doi:10.1186/s40249-022-01010-x)

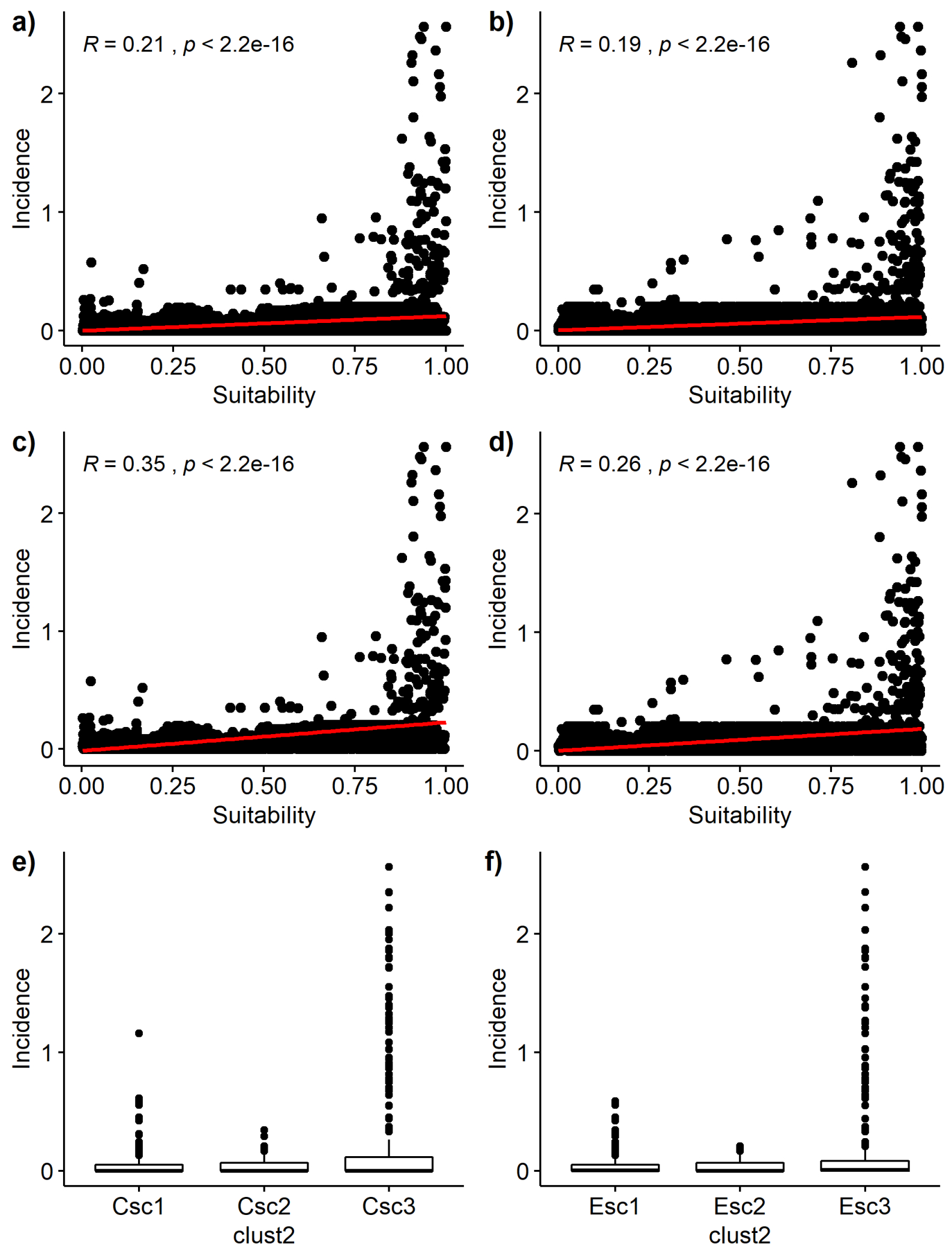

Supplement: Supplementary file 1 — Additional file 1. Comparisons between human leptospirosis incidence rate in the Urban agglomeration of Santa Fe and the environmental and socio-economic suitability (“suitability”) and suitability profiles (“clusters”) for the disease. References: Pearson correlation between the suitability for the disease and human leptospirosis incidence rate using all incidence data for the Conservative scenario (a) and the Explorative scenario (b). Pearson correlation between the suitability for the disease and human leptospirosis incidence rate using non-null incidence data for the Conservative scenario (c) and the Explorative scenario (d). Boxplot of human leptospirosis incidence rate in the clusters for the Conservative scenario (e) and the Explorative scenario (f). [file 40249_2022_1010_MOESM1_ESM.tiff]
